# Topical Ocular Delivery of TGF-β1 to the Back of the Eye: Implications in Age-Related Neurodegenerative Diseases

**DOI:** 10.3390/ijms18102076

**Published:** 2017-09-30

**Authors:** Chiara Bianca Maria Platania, Vincenzo Fisichella, Annamaria Fidilio, Federica Geraci, Francesca Lazzara, Gian Marco Leggio, Salvatore Salomone, Filippo Drago, Rosario Pignatello, Filippo Caraci, Claudio Bucolo

**Affiliations:** 1 Section of Pharmacology, Department of Biomedical and Biotechnological Sciences, School of Medicine, University of Catania, 95123 Catania, Italy; chiara.platania@unict.it (C.B.M.P.); vincifisi@hotmail.com (V.F.); annafidilio@yahoo.it (A.F.); rica2203@hotmail.it (F.G.); f.lazzara@hotmail.it (F.L.); gmleggio@me.com (G.M.L.); salomone@unict.it (S.S.); f.drago@unict.it (F.D.); 2 Center for Research in Ocular Pharmacology—CERFO University of Catania, 95123 Catania, Italy; r.pignatello@unict.it; 3 Department of Drug Sciences, University of Catania, 95125 Catania, Italy; carafil@hotmail.com; 4 NANO-i—Research Center on Ocular Nanotechnology, University of Catania, 95125 Catania, Italy; 5 IRCSS Associazione Oasi Maria S.S., Institute for Research on Mental Retardation and Brain Aging, 94018 Troina, Italy

**Keywords:** TGF-β1, age-related neurodegenerative diseases, retina, liposomes

## Abstract

Dysregulation of the transforming growth factor-β1 (TGF-β1)/selected small mother against decapentaplegic (SMAD) pathway can be implicated in development of age-related macular degeneration (AMD), and the delivery of TGF-β1 could be beneficial for AMD. We developed a new ophthalmic formulation of TGF-β1 assessing the ocular pharmacokinetic profile of TGF-β1 in the rabbit eye. Small unilamellar vesicles (SUV) loaded with TGF-β1 were complemented with Annexin V and Ca^2+^, and the vitreous bioavailability of TGF-β1 was assessed after topical ocular administration by a commercial ELISA kit. We detected high levels of TGF-β1 (C_max_ 114.7 ± 12.40 pg/mL) in the vitreous after 60 min (T_max_) from the topical application of the liposomal suspension. Ocular tolerability was also assessed by a modified Draize’s test. The new formulation was well tolerated. In conclusion, we demonstrated that the novel formulation was able to deliver remarkable levels of TGF-β1 into the back of the eye after topical administration. Indeed, this TGF-β1 delivery system may be useful in clinical practice to manage ophthalmic conditions such as age-related macular degeneration, skipping invasive intraocular injections.

## 1. Introduction

Transforming growth factor-β1 (TGF-β1) is a member of the TGF-β superfamily, which includes several groups of highly conserved multifunctional cell-cell signalling proteins, controlling cell growth and differentiation, as well as immune suppression and repair after injury [1]. Within the mammalian TGF-β superfamily, TGF-β1, 2, and 3 are important modulators of cell survival and apoptosis [2]. All three TGF-βs are synthesized as homodimeric pro-proteins (pro-TGF-β), cleaved at intracellular level by furin into a larger C-terminal pro-region (latency-associated peptide, LAP), and a shorter N-terminal active peptide, which forms the mature homodimer (25-kDa). The mature 25-kDa TGF-βdimer remains non-covalently associated with LAP before secretion [3,4]. In the central nervous system (CNS), TGF-β2 and three isoforms account for almost all the TGF-β immunoreactivity, while TGF-β1 expression is limited to meninges and choroid plexus. Interestingly, TGF-β1 expression and release increase significantly in response to CNS lesions [5]. Recently, a specific impairment of the TGF-β1 signaling pathway has been demonstrated in Alzheimer’s disease (AD), an amyloid-related neurodegenerative disorder, that shares similar features with age-related macular degeneration (AMD) [6,7,8]. The deficiency of TGF-β1 signaling increases both amyloid-β (Aβ) accumulation and Aβ-induced neurodegeneration in AD models [9]. Levels of TGF-β1 and small latent TGF-β1 decrease in the serum of AD patients [10,11]. The potential use of growth factors to treat AD was extensively reviewed by Lauzon et al. in 2015 [12]. Growing evidence from in vitro and in vivo models of AD indicates a neuroprotective role of TGF-β1 against Aβ toxicity [9,13,14]. Altogether, these studies support the hypothesis that either drugs capable of inducing TGF-β1 secretion or TGF-β1 itself can be neuroprotective. Based on these premises, the rescue of TGF-β1 signaling might represent a new strategy to promote neuroprotection in AD as well as in other amyloid-related neurodegenerative disorders, including AMD. Consistent with this view, we recently found that microRNAs related to the TGF-β pathway are dysregulated in the serum of patients with AMD as well as in the retina and serum of rats that received intravitreal injection of Aβ oligomers, an animal model of AMD [15].

We previously reported that human recombinant TGF-β1 is able to prevent retinal damage elicited by Aβ oligomers [8]; in this study, we injected human Aβ_1–42_ oligomers into the rat’s eye, with or without TGF-β1 treatment; co-injection of TGF-β1 significantly protected the rat retina from Aβ-induced damage. This was the first evidence that TGF-β1 could be useful in clinical practice to preserve retinal damage. However, the intravitreal injection represents an invasive route of drug administration, though it is currently used by ophthalmologists to deliver anti-vascular endothelial growth factor (anti-VEGF) drugs and corticosteroids. Intravitreal administration has some drawbacks, such as patient discomfort, especially when multiple injections are required, as well as an association with the risk of endophthalmitis [16]. Furthermore, systemic delivery of TGF-β1 has potential drawbacks due to its fibrogenic activity and its pathogenetic role in idiopathic pulmonary fibrosis [17]. Drug delivery to the back of the eye through topical administration is challenging, especially for biologics [18]; however, several strategies have been carried out so far [19,20,21,22,23]. Therefore, the aim of our study was to figure out whether TGF-β1 is able to reach the back of the eye after topical administration of the liposomal formulation. The employed strategy followed the work of Davis et al. [24]; they tested negatively charged small unilamellar vesicles loaded with bevacizumab and complemented with Annexin V and Ca^2+^ prior topical ocular administration. This strategy was developed by Davis et al. [24] on the basis of the following evidences: (i) high concentration of Annexin V was found at the corneal epithelium [25], indicating high ocular biocompatibility; and (ii) Annexin V is able to bind phosphatidyl serine of the cell membrane, which can be crossed by this protein in the presence of Ca^2+^ [26,27,28,29].

## 2. Results

### 2.1. Small Unilamellar Vesicles Preparation and Characterization

**Figure 1:**
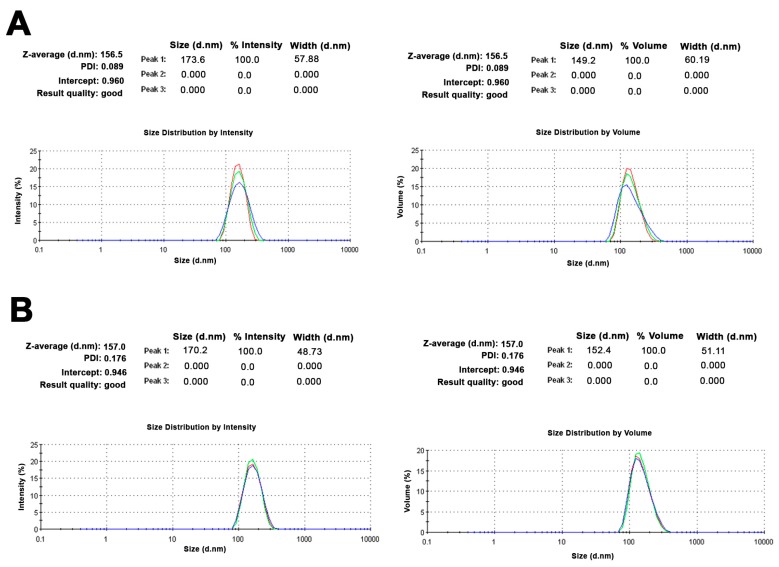
Results of dynamic light scattering (DLS) analysis for transforming growth factor-β1 (TGF-β1)-loaded unilamellar vesicles (SUVs) before (**A**) and after the addition of Annexin V (**B**). Mean particle size results for each sample were expressed by signal intensity (**left**) or by volume (**right**).

**Figure 2:**
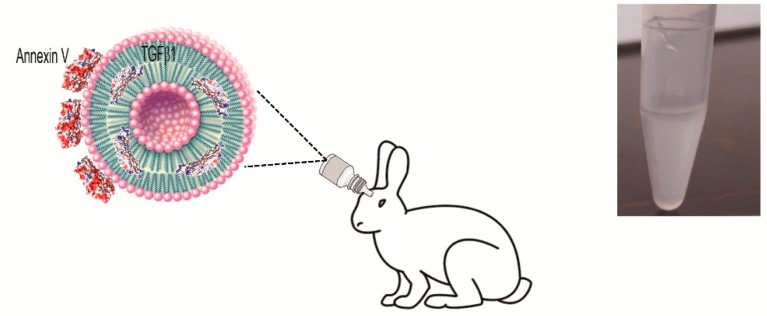
Description of topical liposomal suspension loaded with TGF-β1.

**Figure 3:**
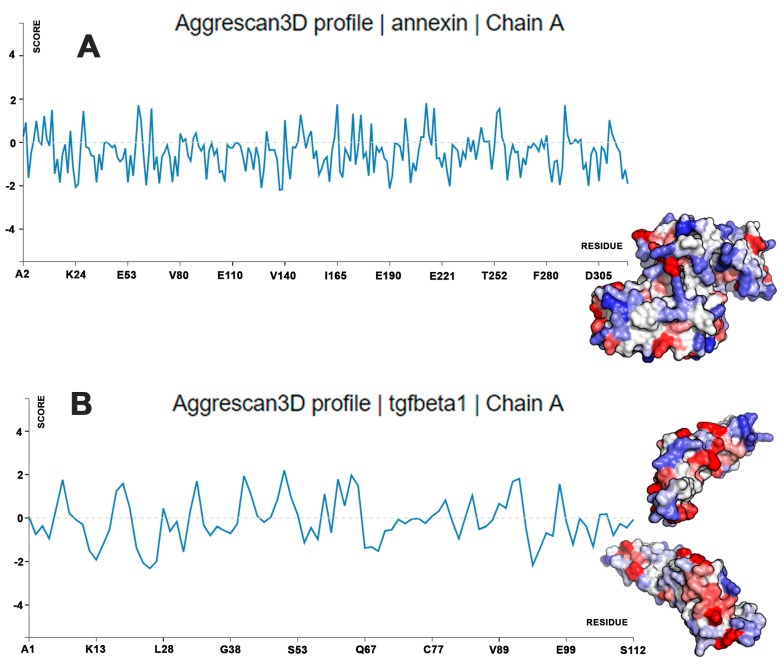
Aggrescan3D calculations: aggregation propensity and water solubility of Annexin V (**A**) and TGF-β1 (**B**). The Aggrescan3D profile shows the score for each residue at the protein surface; the more negative the score, the lower the contribution of the residue to protein aggregation. Color code: red residues are predicted to have the highest aggregation propensity and lowest water solubility, while white and blue residues are predicted to have the lowest aggregation propensity and highest water solubility.

Small unilamellar vesicles (SUVs) were formulated with 1,2-dipalmitoyl-*sn*-glycero-3-phosphocholine (DPPC), 1,2-dioleoyl-*sn*-glycero-3-phospho-l-serine (DOPS), and cholesterol (Chol) at fixed concentrations (i.e., 60:15:25 molar ratio, respectively), as previously described [24]. Using a classical thin layer evaporation (TLE) method, a heterogeneous population of multilamellar lipid vesicles (MLV) was obtained, with a mean size above 2 µm. TGF-β1 loading was carried out with the hydration step of a lipid film. Extrusion of MLV through polycarbonate membranes led to the formation of SUVs with an average size of 156 nm and a very high size homogeneity (polydispersity index (PDI) = 0.089) (Figure 1A). The ζ potential value was found to be markedly negative for the SUVs containing the negatively charged DOPS (−16.6 ± 1.2 mV). Measurement of the Ζ potential before and after the extrusion of MLV to form the SUVs did not cause any changes, confirming that this physical process did not affect the mean composition of the vesicles. After the addition of Annexin V and Ca^2+^, the SUV samples did not show relevant changes in the mean size (157 nm; PDI = 0.176) (Figure 1B), while the surface charge became more negative (−28.83 ± 0.9 mV), suggesting that Annexin V remained located on the external surface of the phospholipid vesicles (Figure 2). This hypothesis was supported by the calculation of protein stability in water and aggregation propensity, calculated with Aggrescan3D for Annexin V and TGF-β1 [30]. Annexin V was predicted to have lower aggregation propensity and higher water solubility (Aggrescan3D score −102.96) compared to TGF-β1 (Aggrescan3D score −11.19). Therefore, TGF-β1 is likely embedded in the lipid matrix during SUV preparation, and Annexin V interacts with the SUV surface (Figure 2 and Figure 3). Additionally, as shown in Figure 3, TGF-β1 showed more hot-spot residues (red color) with high aggregation propensity in comparison to Annexin V. TGF-β1 encapsulation efficiency in the extruded SUVs was 30%.

### 2.2. Vitreous Availability and Ocular Tolerability of TGF-β1 Eye Drops

**Figure 4:**
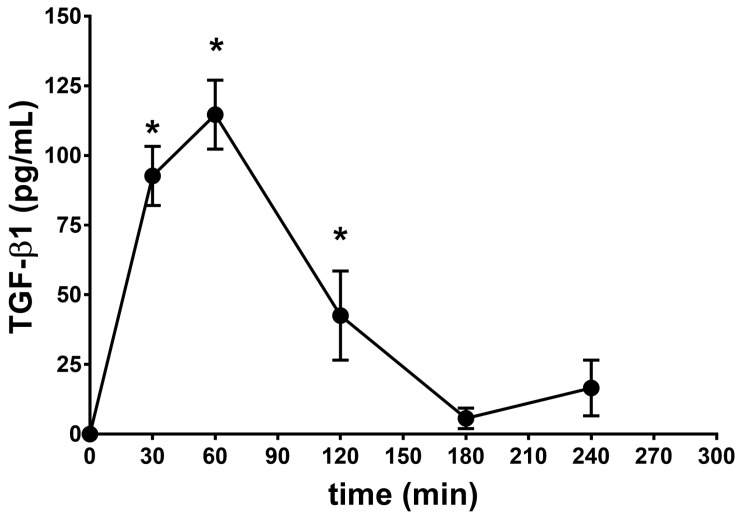
Bioavailability of the novel TGF-β1 formulation in rabbit vitreous. * *p* < 0.01 vs. basal level (control animals) (35.60 ± 10 pg/mL). The curve was normalized to TGF-β1 basal levels.

In order to obtain a pharmacokinetic profile of the TGF-β1 ophthalmic formulation ([TGF-β1] = 125 ng/mL), 30 μl of the TGF-β1/AnnexinV-Ca^2+^/DOPS-SUV liposomal suspension was administered to rabbit eye, two times within 5 min from the first administration (7.5 ng of TGF-β1 per eye). After 30, 60, 120, 180, and 240 min from TGF-β1 administration, the rabbits were sacrificed in order to remove the eye and collect the vitreous. TGF-β1 in the vitreous was measured with a commercial ELISA kit (see Methods section). We followed the protocol of the ELISA kit with the exception of the acidification step, in order to measure the administered TGF-β1 and not the endogenous growth factor. The pharmacokinetic profile generated in the present study (Figure 4) demonstrated remarkable levels of TGF-β1 in the back of the eye (AUC_0–240min_ was 11,331 ± 894 pg × min/mL) that received SUVs loaded with TGF-β1 (Table 1). In particular, we detected high levels of TGF-β1 (C_max_ 114.7 ± 12.40 pg/mL) in the vitreous 60 min (T_max_) after topical application of the eye drops. These levels of TGF-β1 are consistent with a dose previously found effective in a model of AMD [8]. A separate set of animals (control animals) received eye drops formulated with unloaded SUVs (with no TGF-β1) in order to assess basal vitreous levels of TGF-β1 (35.60 ± 10 pg/mL) and to assess vehicle ocular tolerability (Table 2).

The ocular tolerability of the TGF-β1 eye drops formulation was assessed by a modified Draize’s test in a separate set of animals. We found that TGF-β1 eye drops were well tolerated by rabbits and the score for each parameter was zero at all times of observation (Table 2).

## 3. Discussion

TGF-β1 acts through a receptor complex constituted by the serine/threonine kinase ALK/TGF-β type I receptor and TGF-β type II receptor (TβRII). TGF-β1 binding to TGF-β type II receptor induces the assembly of type I and type II receptors into a complex, with the subsequent transphosphorylation of type I receptor by the type II receptor kinase. The subsequent activation of type I receptor leads to the phosphorylation of selected small mother against decapentaplegic (SMAD) proteins which, in turn, translocate into the nucleus in order to activate the expression of different target genes involved in cell proliferation and survival [1,9]. Besides SMAD-mediated gene transcription, TGF-β1 activates SMAD-independent pathways, including the extracellular-regulated kinase (ERK) pathway [31] and the phosphatidylinositide 3-kinases(PI3K)/ protein kinase B (Akt) pathway [8,32]. 

TGF-β1 exerts neuroprotective effects in experimental models of neurodegenerative disorders [9,10] and is protective for retinal ganglion cells [33]. Furthermore, TGF-β1 signaling is essential for maintaining the integrity of the blood-retinal barrier and blood-brain barrier [34]. Previous studies from our lab indicated that intravitreal injection of TGF-β1 may protect retinal tissue in a rat model of AMD [8]. In particular, we found that intravitreal injection of Aβ induced a strong increase of BCL2-Associated X Protein (Bax), a proapoptotic protein, and reduced the antiapoptotic protein B-cell lymphoma 2 (Bcl-2). Intravitreal injection of TGF-β1 decreased the Bax/Bcl-2 ratio, while the beneficial effect of TGF-β1 inhibition of the kinase ALK/TGF-β type I receptor counteracted the protective effect of TGF-β1. From this finding, in view of developing a TGF-β1-based treatment for AMD, we moved forward to explore the pharmacokinetics profile of a topical ophthalmic formulation of TGF-β1.

Neurotrophic factor therapy represents a tough challenge for CNS drug discovery, because protein growth factors do not cross the blood–brain barrier and require intracerebral administration to be effective. The eye is considered an extension of brain and the retina is part of the CNS; therefore, retina drug delivery of neurotrophic factors brings challenges somehow similar to CNS drug delivery. Intravitreal injections are commonly used in clinical practice for drug delivery to the posterior segment of the eye, despite the risks of such an invasive maneuver. 

The ocular availability of a large protein, the anti-VEGF bevacizumab, has been recently increased through an innovative strategy. Davis et al. [24] developed a topical formulation of the monoclonal antibody bevacizumab (149 kDa) encapsulated in SUVs containing phosphatidylserine, with Annexin V and Ca^2+^ extemporaneously added to the topical formulation [24]. The authors exploited the ability of Annexin V to cross the corneal epithelium cell membrane in the presence of Ca^2+^ [26,27,29,35] and achieved a remarkable ocular bioavailability of bevacizumab. Annexin V is adsorbed at the SUV surface; thus, while crossing the cell membrane, Annexin V enhances SUV uptake [24].

We carried out a similar protocol, by developing a formulation of TGF-β1 encapsulated in SUVs, supplemented with Annexin V and Ca^2+^ prior to topical application to a rabbit’s eye [24]. The pharmacokinetics profile we obtained showed a remarkable ocular bioavailability of TGF-β1 following a single administration of our liposomal formulation. Worthy of note, TGF-β1 levels detected in the present study are consistent with levels obtainable following intravitreal injection of an active pharmacological dose [8]. These findings may have relevant clinical implications when considering that intravitreal injections are invasive and risky for the patients. Moreover, the TGF-β1 eye drops were well tolerated, increasing the potential use in clinical practice. 

## 4. Material and methods

### 4.1. Unilamellar Vesicle Preparation

DPPC was purchased from Genzyme Pharmaceuticals, Liestal, Switzerland; DOPS were from Avanti Polar Lipids, Alabaster, AL, USA. Cholesterol (Chol); and phosphate-buffered saline (PBS) (tablets, pH 7.4) was purchased from Sigma-Aldrich (Milan, Italy); methanol was a product ordered from Riedel-DeHaёn (Seelze, Germany), and chloroform was purchased from VWR PBI International (Milan, Italy). Solvents were used as received. The multilamellar vesicle (MLV) liposomal suspensions were obtained by hydration of a phospholipid film using the thin layer evaporation (TLE) method [36]. Negatively charged vesicles, consisting of 1,2-dipalmitoyl-*sn*-glycero-3-phosphocholine (DPPC), 1,2-dioleoyl-*sn*-glycero-3-phospho-l-serine (DOPS), and cholesterol (Chol) at fixed concentrations (DPPC-DOPS-Chol) in a 60:15:25 molar ratio were produced, as previously described [24]. The lipids (1 mg total) were placed in a test tube and dissolved in 1 mL of a 1:1 (*v/v*) chloroform-methanol mixture. The solution was evaporated to dryness under a nitrogen stream and slow rotation, forming a thin lipid film at the bottom of the tube. To remove all the residual solvents, the tubes were placed in a Büchi T-50 oven at 30 °C under high vacuum for 6–8 h. The hydration process was accomplished by adding to the lipid film 2 mL of PBS (pH 7.4) (total volume) containing 250 ng/mL of TGF-β1 (human recombinant TGF-β1 cod. 240-B-010, R&D Systems Inc., USA). The tube was heated in a water bath at 50 °C for 2 min under mild heating; the entire procedure was repeated three times. After hydration, samples were left to equilibrate at room temperature for 2 h. The MLV suspension was turned into a small unilamellar vesicles (SUV) preparation by membrane extrusion, using a LiposofastTM system (Avestin, Ottawa, ON, Canada). Each MLV sample was sequentially passed through two stacked polycarbonate membranes, with a nominal pore diameter of 400 nm and then 100 nm, pushing the suspension back and forth between two gastight glass syringes 19 times at room temperature. To determine the amount of encapsulated TGF-β1, a 0.5-mL fraction of SUV suspension was loaded into a packed 1 × 8 cm glass column filled with Sephadex G-25 (mean bead size: 50–150 mm) (Sigma, St. Louis, MO, USA) and eluted with 0.13 M phosphate-buffered solution, pH 7.4. The fractions (opaque) containing the liposomes were pooled, treated with the assay buffer used for ELISA quantification, and filtered through a 0.22-nm nylon 13-mm filter (Whatman International Ltd., Maidstone, UK). TGF-β1 content was then analyzed using the ELISA kit ADI-900-155 (Enzo Life Bioscience, Farmingdale, NY, USA). The final drug concentration in the SUV was expressed as the entrapment efficiency, corresponding to the percentage of drug remaining encapsulated in the liposomes versus the amount initially added; TGF-β1 encapsulation efficiency was found to be 30%. Unloaded SUVs were prepared similarly for the biological experiments, using pure PBS as a hydration medium. Each liposomal batch was characterized within 24 h from preparation. The electrophoretic mobility and ζ potential were determined by a particle electrophoresis analyzer (Zetasizer Nano ZS90, Malvern, UK). The apparatus consisted of a He-Ne laser with a maximal power of 4 mW, at a wavelength of 633 nm. Each sample was diluted 1:100 with HPLC-grade water for the test. Up to 100 measurements on each sample were recorded at room temperature to calculate the electrophoretic mobility and the corresponding ζ potential values, by using a Smoluchowski constant (*K*a) value of 1.5. The mean size (Z-ave) and polydispersity index (PDI) were determined by dynamic light scattering using the same instrument. Samples were 10-fold diluted with HPLC-grade water before the analysis. The collected values are the means ± SD of 90 measurements (three sets of 10 measurements in triplicate). Soon before ocular topical administration, recombinant human Annexin V (#1005 purchased by BioVision, Milpitas, CA, USA) and CaCl_2_ (purchased by Sigma-Aldrich, Milan, Italy) were added to the TGF-β1 liposomal suspension. The TGF-β1 final concentration was 125 ng/mL, Annexin V was added to a final concentration of 15 μg/mL in order to obtain an average of 30 molecules per vesicle, as reported and calculated by Davis et al. [24], and CaCl_2_ was added in order to obtain 2 mM as the final concentration [24].

### 4.2. Calculations of Protein Aggregation Propensity and Water Stability 

Prediction of localization of Annexin V and TGF-β1 at the surface or lipid matrix of SUVs was carried out with Aggrescan3D [30]. The more negative the Aggrescan3D score, the more soluble the protein and the lower its aggregation propensity. Furthermore, Aggrescan3D provides information about hot-spot amino acid residues involved in protein aggregation and indeed stability in water. In order to carry out protein aggregation propensity, the protein data bank (PDB) files 1KLC and 2XO2 were uploaded into the Aggresca3D server, accounting for TGF-β1 and Annexin V X-ray structures, respectively.

### 4.3. In Vivo Study

New Zealand albino rabbits were purchased by Envigo (Udine, Italy). Rabbits, weighing approximately 2–2.2 Kg, were housed for one week prior the study, while they were fed on standard laboratory food and allowed free access to water in a room with standard temperature and humidity conditions according to a 12-h light/12-h dark cycle. Thirty microliters of the TGF-β1 liposomal formulation was topically administered to the rabbit eye, two times within 5 min from the first administration. After 30, 60, 120, 180, and 240 min from TGF-β1 administration, rabbits were killed by intravenous injection of Tanax (0.3 mL/kg; Intervet Italia, Milano, Italy). After the rabbits were euthanatized, vitreous (0.2 mL) was aspirated with a 25-gauge needle attached to a 5-mL disposable syringe, driven at 3.0 mm from limbus, and guided toward the center of the eyeball; care was taken to avoid bleeding when the needle was introduced (vitreous samples were stored at −80 °C until analysis) [37,38]. Housing and treatments were in accordance with the Association for Research in Vision and Ophthalmology (ARVO) Statement for the Use of Animals in Ophthalmic and Visual Research. Rabbits were randomly distributed in each group. The number of animals required for the study was reduced following the 3R (replace, reduce, and refinement) guide for animal research. This design still provides statistical significance.

### 4.4. TGF-β1 Measurements in Rabbit Vitreous

Vitreous samples were sonicated for 5 min in an ice-water bath, and TGF-β1 levels were measured by means of a commercial ELISA kit (ADI-900-155, Enzo Life Bioscience, Farmingdale, NY, USA) as previously reported [39]. The sensitivity of the ELISA kit, as reported in the data sheet, is 3.3 pg/mL. In order to measure only the exogenous TGF-β1, we avoided the acidification protocol that leads to the dissociation of the LAP/ TGF-β1 endogenous complex. TGF-β1 levels were measured in the vitreous of rabbits that were sacrificed 30, 60, 120, 180, and 240 min (*n* = 4/time point) after ocular topical administration of TGF-β1 formulation (7.5 ng of TGF-β1 per eye). Those levels were compared to TGF-β1 levels detected in the vitreous of rabbits that received unloaded SUVs. Each measurement on each sample was carried out three times. PK parameters (C_max_, T_max_, AUC_0–240min_, λz, apparent elimination half-life) were calculated with GraphPad Prism^®^ (La Jolla, CA USA) [40]. 

### 4.5. Ocular Tolerability Assessment

A separate set of animals was used to assess ocular tolerability. Animals were randomly assigned to SUV-TGF-β1 (*n* = 4) and to unloaded SUV treatment groups (*n* = 4). The potential ocular irritancy and/or damaging effects of the formulation were evaluated according to a modified Draize’s test [41]. A slit lamp (mod. 4179T Sbisà, Florence, Italy) was used. Congestion, swelling, and discharge of the conjunctiva were graded on a scale from 0 to 3 (0 = normal; 1, 2, and 3 = discrete, moderate, and intense dilatation of conjunctival vessels, respectively), 0 to 4 (0 = normal; 1, 2, 3, and 4 = discrete, moderate, intense, intense + lid closure conjunctival swelling, respectively), and 0 to 3 (0 = normal; 1, 2, and 3 = discrete, moderate, and intense discharge, respectively). Iris hyperemia was graded on a scale from 0 to 4 (0 = normal, 1 = discrete dilatation of iris vessels; 2 = moderate dilatation of iris vessels; 3 = intense iridal hyperemia with flare in the anterior chamber; 4 = intense iridal hyperemia with flare in the anterior chamber and presence of fibrinous exudates). Corneal opacity was graded on a scale from 0 to 4 (0 = normal, 1 = some spots of opacity; 2 = diffuse cortical opacity; 3 = cortical and nuclear opacity; 4 = intense opacity plus posterior subcapsular opacity). Eye drops (30 μL) were topically administered in the right eye every 30 min for 6 h (12 treatments). At the end of the treatment, two observations at 10 min and 6 h were carried out to evaluate the ocular tissues. Observations were made by two independent, masked observers. Methylene blue staining was used to evaluate the corneal integrity, which allows an accurate determination of the extent of epithelial damage because of its poor diffusion through the stroma layer of the cornea.

### 4.6. Statistical Analysis

Operators were blind to treatment groups. All data were expressed as means ± standard deviation. Statistical analysis was conducted using t-test followed by ANOVA. In all cases, statistical significance was set at *p* < 0.01. Statistical analysis was carried out with GraphPad Prism ^®^ (La Jolla, CA, USA).

## 5. Conclusions

In conclusion, we demonstrated that the liposomal ophthalmic formulation based on DPPC, DOPS, and Chol (60:15:25 molar ratio) was able to deliver a significant amount of TGF-β1 into the back of the eye, by exploiting the ability of Annexin V to cross the corneal epithelium cell membrane in the presence of Ca^2+^. Therefore, this formulation may be useful in clinical practice to manage ophthalmic conditions such as AMD, because it does not require invasive intraocular injections of TGF-β1.

## Figures and Tables

**Table 1:** Pharmacokinetics (PK )parameters.

Formulation	λz	* *t*_½_ (h)	C_max_ (pg/mL)	T_max_ (min)	AUC_0–240min_ (pg × min/mL)
SUV-TGF-β1	0.90	0.77	114.7 ± 12.4	60	11,331 ± 894

* Apparent elimination half-life (*t*_1/2_) was calculated as follows: 0.693/λz.

**Table 2:** Ocular tolerability of small unilamellar vesicles delivering TGF-β1.

Formulation	Conjunctiva			Iris Hyperemia	Corneal Opacity
	Congestion	Swelling	Discharge		
SUV unloaded (10 min)	0	0	0	0	0
SUV unloaded (6 h)	0	0	0	0	0
SUV-TGF-β1 (10 min)	0	0	0	0	0
SUV-TGF-β1 (6 h)	0	0	0	0	0
